# Improved One-Way Hash Chain and Revocation Polynomial-Based Self-Healing Group Key Distribution Schemes in Resource-Constrained Wireless Networks

**DOI:** 10.3390/s141224358

**Published:** 2014-12-18

**Authors:** Huifang Chen, Lei Xie

**Affiliations:** 1 Department of Information Science & Electronic Engineering, Zhejiang University, Hangzhou 310027, China; E-Mail: xiel@zju.edu.cn; 2 Zhejiang Provincial Key Laboratory of Information Network Technology, Hangzhou 310027, China

**Keywords:** wireless networks, group communication, group key distribution, self-healing, collusion attack

## Abstract

Self-healing group key distribution (SGKD) aims to deal with the key distribution problem over an unreliable wireless network. In this paper, we investigate the SGKD issue in resource-constrained wireless networks. We propose two improved SGKD schemes using the one-way hash chain (OHC) and the revocation polynomial (RP), the OHC&RP-SGKD schemes. In the proposed OHC&RP-SGKD schemes, by introducing the unique session identifier and binding the joining time with the capability of recovering previous session keys, the problem of the collusion attack between revoked users and new joined users in existing hash chain-based SGKD schemes is resolved. Moreover, novel methods for utilizing the one-way hash chain and constructing the personal secret, the revocation polynomial and the key updating broadcast packet are presented. Hence, the proposed OHC&RP-SGKD schemes eliminate the limitation of the maximum allowed number of revoked users on the maximum allowed number of sessions, increase the maximum allowed number of revoked/colluding users, and reduce the redundancy in the key updating broadcast packet. Performance analysis and simulation results show that the proposed OHC&RP-SGKD schemes are practical for resource-constrained wireless networks in bad environments, where a strong collusion attack resistance is required and many users could be revoked.

## 1. Introduction

Many applications of wireless networks require secure group communications, especially in a hostile environment. In order to protect the sensitive data, group communication keys (also named as group session keys) could be used to encrypt exchanged messages among communicating group members. Therefore, the group key management is critical for providing secure communications.

However, providing efficient key distribution in resource-constrained wireless networks, such as wireless sensor networks, is a challenging issue due to some characteristics of wireless networks.

First, a legitimate group member may not receive the key broadcast message for a particular session due to the unreliable wireless medium, which makes the user request the group manager (GM) to re-transmit the message. When the group size is large, re-transmissions could overwhelm the GM potentially. Furthermore, in some applications with high security requirement, it is important that users only transmit essential messages to avoid making themselves vulnerable. It is desirable to have the self-healing property that enables legitimate group members to recover lost session keys on their own, instead of requesting additional transmissions from the GM.

Second, users may join and/or leave the group frequently. For a large communication group, the group session keys have to be updated due to dynamic group members, which result in the network resource consumption. Hence, an efficient node revocation and join mechanism is important for dynamic communication groups.

Third, wireless devices have limited computation capability, memory and energy. Using energy-consuming techniques, such as the public-key cryptography, to realize the group key management is not applicable for resource-constrained wireless networks. Hence, the energy-efficient property is required.

Three articles [1–3], reviewing self-healing group key distribution (SGKD) schemes have appeared in the literature. Tian *et al.* in [1] provides a survey of available solutions, which is focused on the possible scheme extensions, such as sponsorization or mutual-healing. In [2], the author analyzes the practicality of SGKD schemes in the resource-constrained wireless sensor networks. This review is focused on the scheme performance in terms of the communication overhead and storage overhead. In [3], authors identified three building blocks of the SGKD scheme, selective key distribution mechanism, pre-distributed secret data management and self-healing mechanism, to classify and compare the existing solutions. Based on this three-dimensional classification, a comprehensive review of the development in the area of SGKD schemes is provided.

### 1.1. Previous Work

Staddon *et al.* first introduced the concept of the self-healing group key distribution (SGKD), and proposed a non-interactive and reliable key distribution scheme in [4]. The basic idea of the SGKD is to broadcast information that is useful only for legitimate users. In this scheme, users use the secret sharing to bind the capability of recovering lost session keys with the membership. Combined with pre-distributed secrets, legitimate users can recover a session key; otherwise, revoked users cannot infer useful information. However, this scheme has high storage and communication overheads.

Based on the work in [4], several improved SGKD schemes have been proposed [5–29]. In order to increase the efficiency of the scheme in [4], Liu *et al.* proposed some new schemes by combining a personal secret distribution technique with self-healing [5]. Blundo *et al.* analyzed the security model defined in [4,5], and found that it is impossible to satisfy all of the security requirements. Then, based on the self-healing technique with a slightly modified framework in [6] and the self-healing mechanism in [7], a novel SGKD scheme enabling a user to recover all previous session keys from a single key broadcast message was proposed. Hong and Kang proposed a revocation polynomial-based SGKD scheme (RP-SGKD) with low storage and communication overheads [8].

Recent, many hash chain-based SGKD (HC-SGKD) schemes, one-way hash chain (OHC) and dual directional hash chain (DDHC), were proposed in [9–16]. Due to the efficiency of the hash function, these HC-SGKD schemes reduce communication and storage overheads obviously. However, the performance improvement is at the cost of the property of the collusion attack resistance. That is, revoked users colluding with new joined users can recover all session keys, which they are not entitled to get [1].

In [17–19], the pre-arranged life cycle-based SGKD schemes were proposed to make those HC-SGKD schemes resist to the collusion attack. However, these schemes can only apply to the scenario in which the user's life cycle is pre-determined, and the collusion of revoked users within the life cycles and new joined users can recover unauthorized session keys.

In order to resolve the collusion attack resistance problem in existing HC-SGKD schemes, we proposed an SGKD scheme based on the one-way hash chain and revocation polynomial for wireless sensor networks in [20]. However, as using the personal secret structure in Dutta *et al.*'s scheme, the RP-SGKD scheme proposed in [20] inherits the limitation of SGKD schemes in [10,11]. That is, the maximum allowed number of sessions should not be larger than the maximum number of revoked users.

Other techniques, such as subset difference re-keying [21], bilinear pairings [22,23], vector space secret sharing [24,25] and the exponential arithmetic [26], are also used to design SGKD schemes.

Among those existing SGKD schemes, the polynomial secret sharing is the most common cryptographic technique used to implement self-healing key distribution [22]. With regard to the construction method, the polynomial is classified into two types, the revocation polynomial and the access polynomial. Both of them guarantee that only legitimate users can recover the session key(s), while illegitimate users cannot. The SGKD schemes in [5,7–20] are based on the revocation polynomial, and schemes in [27–30] are based on the access polynomial.

Moreover, the hash chain, another cryptographic technique, is used to design the SGKD scheme with other cryptographic techniques. The schemes in [9–20] are hash chain and revocation polynomial-based SGKD (HC&RP-SGKD) schemes, and schemes in [29,30] are hash chain and access polynomial-based SGKD (HC&AP-SGKD) schemes.

### 1.2. Problems in Existing RP-SGKD Schemes

In this paper, we focus on the SGKD scheme based on the revocation polynomial. After investigating existing RP-SGKD schemes, we find that, except for the collusion attack resistance problem in the HC-SGKD schemes, three other common weaknesses for existing RP-SGKD schemes need to be resolved.

First, the maximum allowed number of revoked/colluding users is limited to be *t*, where *t* is the degree of the personal secret polynomial.

Second, the redundancy exists in the key updating broadcast packet, and the communication overhead increases quickly along with the number of sessions.

Third, given the size of the session key updating broadcast packet, the maximum allowed number of sessions and revoked users is too small to use these existing schemes in real resource-constrained wireless networks.

Although the collusion attack resistance problem is partially resolved in [20], the problem, that the maximum allowed number of sessions is limited by the maximum number of revoked users, still exists.

### 1.3. Our Contributions

Two improved SGKD schemes using the one-way hash chain (OHC) and revocation polynomial in resource-constrained wireless networks are proposed. In the proposed SGKD schemes, by binding the time at which the user joins the group with its capability of recovering group session key(s), some novel methods are presented to utilize one-way hash chain, and to construct the personal secret, the revocation polynomial and the key updating broadcast packet.

To solve the collusion attack resistance problem in existing HC-SGKD schemes and eliminate the limitation of the maximum number of revoked user on the maximum allowed number of sessions, we propose the first SGKD scheme. However, as same as most existing SGKD schemes in [4–12,20], the storage overhead of each user in the first proposed SGKD scheme is high, and determined by the maximum number of revoked user or the maximum allowed number of sessions. To eliminate the impact of the maximum number of revoked user or the maximum allowed number of sessions on the storage overhead, we further propose the second SGKD scheme, a constant storage overhead scheme, to achieve a good tradeoff between the storage overhead and the communication overhead.

Compared to existing RP-SGKD schemes, the main advantages of the proposed schemes are four-aspect. First, the collusion attack resistance problem in existing HC-SGKD schemes is solved. Second, a stronger security and more colluding users are to be supported under same conditions. Third, the total communication overhead is reduced without increasing the storage overhead. Fourth, the limitation of the maximum number of revoked user on the maximum allowed number of sessions is eliminated in the proposed SGKD schemes. And the storage overhead is constant in the second SGKD scheme.

The remainder of the paper is organized as follows. In Section 2, the security model on which the proposed schemes are based is defined. In Section 3, two improved SGKD schemes are presented, and the improvements and security performance are analyzed. In Section 4, the performance comparison with some existing schemes is given. Finally, we conclude the paper in Section 5.

## 2. Security Model

In this section, we briefly define the security model used in the paper. Notations used in the paper and the corresponding denotations are summarized in Appendix (Table A1).

To clarify the performance of the proposed SGKD schemes, the security model used in this paper is defined as follows.

Suppose a communication group in wireless networks with a GM and a set of group users. Each group member is uniquely identified by an ID number *i*, the group member is denoted as *U_i_*, *i* ϵ {1, 2, …, *N*}, and *N* is the largest ID number. All of the operations perform in a finite field, *F_q_*, where *q* is a prime, and *q* > *N*. The lifetime of the SGKD scheme is partitioned into *m* sessions.

**Definition 1: (self-healing group key distribution with *mt*-revocation capability)** The scheme is a self-healing group key distribution with *mt*-revocation capability if the following conditions are satisfied.

(a). For a legitimate group member *U_i_*, $U_{i}\epsilon G_{j}^{j^{\prime }}$ , 1 ≤ *j′* ≤ *j* ≤ *m*, the session key for session *j*, *K_j_*, is determined by the key updating broadcast packet for session *j*, *B_j_*, and the personal secret, *S_i_*. That is,
  (1) $$H(K_{j}|B_{j},S_{i})=0$$
(b). No information about *K_j_* (1 ≤ *j* ≤ *m*) can be obtained from either key updating broadcast packets or personal secrets only. That is,
  (2) $$H(K_{j}|S_{1},S_{2},\ldots ,S_{N})=H(K_{j}|B_{1},B_{2},\ldots ,B_{m})=H(K_{j})$$
(c). (*mt*-revocation capability) Let **R***_j_* be a set of users be revoked before and in session *j*, ${\text{R}}_{j}=\left\{R_{j}^{1},R_{j}^{2},\ldots ,R_{j}^{j}\right\}$, where $R_{j}^{j^{\prime }}$ is the set of users joined the group in session *j′* and be revoked before or in session *j*, $|R_{j}^{j'}|\leq t$ and |**R***_j_*| ≤ *jt* for 1 ≤ *j* ≤ *m*. The scheme has *mt*-revocation capability if for a given **R***_j_*, the GM can generate a key updating broadcast packet, *B_j_*, in order that *U_i_* who does not belong to **R***_j_* recovers *K_j_*, whereas the revoked user *U_r_*, *U_r_*∈**R***_j_*, cannot recover *K_j_*. That is,
  (3) $$H(K_{j}|B_{j},S_{i})=0,H(K_{j}|B_{j},\{S_{r}|U_{r}\in R_{j}\})=H(K_{j})$$
(d). (Self-healing property) The scheme is self-healing if any user *U_i_*, who joined the group in session *j*_1_ and is still a legitimate group member in session *j*_2_, can recover lost session key for session *j*, *K_j_*, from the key updating broadcast packet for session *j*_2_, *B_j_*_2_, and *j*_1_ < *j* < *j*_2_. That is,
  (4) $$H(K_{j}|B_{j_{2}},\{S_{i}|U_{i}\in G_{j}^{j_{1}}\})=0$$

**Definition 2: (*mt*-wise forward secrecy)** Let **R***_j_* be a set of users be revoked before and in session *j*, ${\text{R}}_{j}=\left\{R_{j}^{1},R_{j}^{2},\ldots ,R_{j}^{j}\right\}$, where $R_{j}^{j^{\prime }}$ is the set of users who joined the group in session *j′* and are revoked before or in session *j*, $|R_{j}^{j'}|\leq t$ and |**R***_j_*| ≤ *jt* for 1 ≤ *j* ≤ *m*. The scheme guarantees *mt*-wise forward secrecy if for any set **R***_j_*, all users in **R***_j_* cannot get any information about *K_j_*_+1_ even with the knowledge of session keys before session *j*. That is,

(5) $$H(K_{j+1}|B_{1},B_{2},\ldots ,B_{m},\{S_{r}|U_{r}\in R_{j}\},K_{1},K_{2},\ldots ,K_{j})=H(K_{j+1})$$

**Definition 3: (*any*-wise backward secrecy)** Let **D***_j_* be the set of users joined the group after session *j*, **D***_j_* = {*D^j^*^+1^, *D^j^*^+2^, …, *D^m^*}, where *D^j^*^′^ (*j* + 1 ≤ *j′* ≤ *m*) is the set of users joined the group in session *j′*, and 1 ≤ *j* ≤ *m*. The scheme guarantees *any*-wise backward secrecy if for any set **D***_j_*, all users in **D***_j_* cannot get any information about *K_j_* even with the knowledge of session keys after session *j*. That is,

(6) $$H(K_{j}|B_{1},B_{2},\ldots ,B_{m},\{S_{v}|U_{v}\in D_{j}\},K_{j+1},K_{j+2}\ldots ,K_{m})=H(K_{j})$$

**Definition 4: (*mt*-wise collusion attack resistance capability)** Let **R***_j_*__1__ be the set of users be revoked before and in session *j*_1_. Let **D***_j_*__2__ be the set of users joined the group after session *j*_2_. The scheme has *mt*-wise collusion attack resistance capability if given any two disjoint sets **R***_j_*__1__ and **D***_j_*__2__ (*j*_1_ < *j*_2_), users in **R***_j_*__1__ colluding with users in **D***_j_*__2__ cannot recover *K_j_* even with the knowledge of {*B*_1_, *B*_2_, …, *B_m_*, {*S_r_*|*U_r_*∈**R***_j_*__1__}} and {*B*_1_, *B*_2_, …, *B_m_*, {*S_v_*|*U_v_*∈**D***_j_*__2__}} for *j*_1_ < *j* ≤ *j*_2_. That is,

(7) $$H(K_{j}|B_{1},B_{2},\ldots ,B_{m},\{S_{i}|U_{i}\in R_{j_{1}}\cup D_{j_{2}}\})=H(K_{j})$$

## 3. Two Improved Self-Healing Group Key Distribution Schemes

### 3.1. The OHC&RP-SGKD Scheme 1

In order to resolve the problems mentioned in Section 1.2, we propose two improved SGKD schemes using the one-way hash chain and the revocation polynomial for resource-constrained wireless networks.

To remove the limitation of the maximum number of revoked user *t* on the maximum allowed number of sessions *m*, *m* < *t* + 1, we change the structure of the personal secret used in [20], and propose the first improved SGKD scheme based on the one-way hash chain and the revocation polynomial, named as the OHC&RP-SGKD scheme 1.

In the proposed OHC&RP-SGKD scheme 1, *m t*-degree polynomials chosen from *F_q_*[*x*], *s*_1_(*x*), *s*_2_(*x*), …, *s_m_*(*x*), are used to replace one 2*t*-degree polynomial in Dutta *et al.*'s scheme and the RP-SGKD scheme in [20]. When joining the group in session *j*, *U_i_* stores *S_i_* = {å*_j_*·*s_j_*(*i*), å*_j_*·*s_j_*_+1_(*i*), …, å*_j_*·*s_m_*(*i*)} as the personal secret, where å*_j_* is the unique session identifier for session *j*. Hence, revealing one or more used secret polynomials has no effect on unused personal secret polynomials, and then it has no effect on following group session keys.

#### 3.1.1. The Scheme Detail

The proposed OHC&RP-SGKD scheme 1, including three phases and two cases, is described as follows.

**Phase 1: Initialization**

The GM independently and randomly chooses *m t*-degree polynomials from *F_q_*[*x*], *s*_1_(*x*), *s*_2_(*x*), …, *s_m_*(*x*), and *m* numbers from *F_q_*, å_1_, å_2_, …, å*_m_*.

Each user *U_i_*, *U_i_* ϵ **G**_1_, receives *S_i_* = {å_1_·*s*_1_(*i*), å_1_·*s*_2_(*i*), …, å_1_·*s_m_*(*i*)} as the personal secret from the GM via a secure communication channel, where **G**_1_ denotes the set of group members at the beginning of session 1.

**Phase 2: Broadcast in Session *j* (1 ≤ *j* ≤ *m*)**

Let **R***_j_* be the set of users be revoked before and in session *j*, ${\text{R}}_{j}=\left\{R_{j}^{1},R_{j}^{2},\ldots ,R_{j}^{j}\right\}$, where $R_{j}^{j^{\prime }}$ is the set of users joining the group in session *j′* and be revoked before or in session *j*, ${\text{R}}_{j}^{j^{\prime }}=\left\{U_{r_{1}^{j^{\prime }}},U_{r_{2}^{j^{\prime }}},\ldots ,U_{r_{w_{j^{\prime }}}^{j^{\prime }}}\right\}$ and $|R_{j}^{j'}|=w_{j^{\prime }}\leq t$. $r_{1}^{j^{\prime }},r_{2}^{j^{\prime }},\ldots ,r_{w_{j^{\prime }}}^{j^{\prime }}$ are the IDs of users in $R_{j}^{j^{\prime }}$. $R_{j}^{j^{\prime }}=\emptyset$ if there are no new joined users in session *j′*.

(1) The GM randomly chooses a number $k_{j}^{0}$ from *F_q_*. And the *j*-th key chain, $\left\{k_{j}^{1},k_{j}^{2},\ldots ,k_{j}^{j}\right\}$, is calculated with one-way hash function, *h*(·), and $k_{j}^{0}$ as follows,
  (8) $$\begin{array}{l} k_{j}^{1}=h(k_{j}^{0})\\ k_{j}^{2}=h(k_{j}^{1})=h(h(k_{j}^{0}))=h^{2}(k_{j}^{0})\\ \ldots \\ k_{j}^{j}=h(k_{j}^{j-1})=h^{2}(k_{j}^{j-2})=\ldots =h^{j}(k_{j}^{0}) \end{array}$$
  For security, $k_{j_{1}}^{0}\neq k_{j_{2}}^{0}$ for *j*_1_ ≠ *j*_2_.
(2) The GM chooses number sets ${R^{\prime }}_{j}^{j^{\prime }}$, ${R^{\prime }}_{j}^{j^{\prime }}=\left\{{r^{\prime }}_{1}^{j^{\prime }},{r^{\prime }}_{2}^{j^{\prime }},\ldots ,{r^{\prime }}_{t-w_{j^{\prime }}}^{j^{\prime }}\right\}$, from *F_q_* for sessions with new joined user(s), where ${r^{\prime }}_{1}^{j^{\prime }},{r^{\prime }}_{2}^{j^{\prime }},\ldots ,{r^{\prime }}_{t-w_{j^{\prime }}}^{j^{\prime }}$ are random numbers, not used as a user ID and different from each other. The GM constructs the revocation polynomials for the users joined the group in different sessions as,
  (9) $$A_{j}^{j'}(x)=\prod_{z=1}^{\left|R_{j}^{j'}\right|}\left(x-r_{z}^{j'}\right)\prod_{z'=1}^{t-|R_{j}^{j'}|}\left(x-r{'}_{z'}^{j'}\right),j'=1,2,\ldots ,j$$
  The purpose of the padding with the elements in ${R^{\prime }}_{j}^{j^{\prime }}$ is to make the constructed revocation polynomials be *t*-degree.
(3) The GM computes
  (10) $$\Phi_{j}^{j'}(x)=A_{j}^{j'}(x)\cdot k_{j}^{j'}+\varepsilon_{j'}\cdot s_{j}(x),j'=1,2,\ldots ,j$$
  where ε*_j′_*·*s_j_*(*x*) and $k_{j}^{j^{\prime }}$ are the masking polynomial and the masking key, respectively.
(4) The GM randomly chooses a session key *K_j_* from *F_q_*.
(5) The GM constructs and broadcasts the message
  (11) $${Β}_{j}={\text{R}}_{j}\cup \text{R}{'}_{j}\cup {\{\Phi }_{j}^{j'}(x)|j'=1,2,\ldots ,j\}\cup \{E_{k_{j}^{j'}}(K_{j'})|j'=1,2,\ldots ,j\}$$
  where ${R^{\prime }}_{j}=\left\{{R^{\prime }}_{j}^{1},{R^{\prime }}_{j}^{2},\ldots ,{R^{\prime }}_{j}^{j}\right\}$.

**Phase 3: Group Session Key Recovery in Session *j* (1 ≤ *j* ≤ *m*)**

When a legitimate group member *U_i_*, $U_{i}\epsilon G_{j}^{j^{\prime }}$, receives *B_j_*, it recovers the group session key via following steps.

(1) *U_i_* evaluates ε*_j′_*·*s_j_*(*i*), $A_{j}^{j^{\prime }}(i)$ and $\Phi_{j}^{j^{\prime }}(i)$, and computes the masking key as
  (12) $$k_{j}^{j'}=\left[\Phi_{j}^{j'}(i)-\varepsilon_{j'}\cdot s_{j}(i)\right]/A_{j}^{j'}(i),j'=1,2,\ldots ,j$$
  $A_{j}^{j^{\prime }}(i)=0$ when $U_{i}\epsilon R_{j}^{j^{\prime }}$, which means that revoked users can recover neither $k_{j}^{j^{\prime }}$ nor *K_j_* from *B_j_*.
(2) *U_i_* computes all masking keys, $\left\{k_{j}^{j^{\prime \prime }}|j^{\prime }\leq j^{\prime \prime }\leq j\right\}$, in the *j*-th key chain with (8).
(3) By decrypting $\left\{E_{k_{j}^{j^{\prime \prime }}}\left(K_{j^{\prime \prime }}\right)|j^{\prime }\leq j^{\prime \prime }\leq j\right\}$ with $\left\{k_{j}^{j^{\prime \prime }}|j^{\prime }\leq j^{\prime \prime }\leq j\right\}$, *U_i_* recovers {*K_j″_*| *j′* ≤ *j″* ≤ *j*}.

**Case 1: Group Member Addition**

If a new user, *U_v_*, joins the communication group in session *j*, a key updating process is launched to ensure the backward secrecy.

The GM allocates *S_v_* = {å*_j_*·*s_j_*(*v*), å*_j_*·*s_j_*_+1_(*v*), …, å*_j_*·*s_m_*(*v*)} as the personal secret to *U_v_* via a secure communication channel. Receiving the personal secret, *U_v_* joins **G***_j_*.

The GM and users in **G***_j_* launch a key updating process, including Phase 2 and Phase 3, to include *U_v_*.

**Case 2: Group Member Revocation**

If a user joined the group in session *j′*, *U_r_*, is revoked in session *j*, a key updating process is launched to ensure the forward secrecy.

The GM includes ( $x-r_{r}^{j^{\prime }}$) into $A_{j^{\prime \prime }}^{j^{\prime }}(x)(j\leq j^{\prime \prime }\leq m)$, which means *U_r_* joins $R_{j^{\prime \prime }}^{j^{\prime }}$ and **R***_j″_*. And then, the GM and users in **G***_j_* launch a key updating process, including Phases 2 and 3, to exclude *U_r_*.

#### 3.1.2. Main Advantages

The proposed OHC&RP-SGKD scheme 1 solves the problems mentioned in Section 1.2, and also has some performance improvements.

(1). With the property of the collusion attack resistance
  In the proposed OHC&RP-SGKD scheme 1, the unique identity for each session is introduced. *U_v_*, who joins the communication group in session *j*, receives *S_v_* = {å*_j_*·*s_j_*(*v*), å*_j_*·*s_j_*_+1_(*v*), …, å*_j_*·*s_m_*(*v*)} as the personal secret, where *å_j_* is the joining time identity for session *j*.
  A user *U_r_*, *U_r_* ∈ **G**_1_, be revoked in session *j*_1_, knows {å_1_·*s_j_*(*r*)| 1 ≤ *j* ≤ *m*}. And *U_v_* joined the group in session *j*_2_ (*j*_1_ *< j*_2_ ≤ *m*) knows {ε*_j_*_2_·*s_j_*(*v*)| *j*_2_ ≤ *j* ≤ *m*}. The collusion of *U_v_* and *U_r_* can obtain {å_1_·*s_j_*(*r*)| 1 ≤ *j* ≤ *m*} and {ε*_j_*_2_·*s_j_*(*v*)| *j*_2_ ≤ *j* ≤ *m*}, but neither {å*_j_*·*s_j_*(*r*)| *j*_1_ < *j* < *j*_2_} nor {å*_j_*·*s_j_*(*v*)| *j*_1_ < *j* < *j*_2_}. Hence, they cannot recover {*K_j_*| *j*_1_ < *j* < *j*_2_}.
  Therefore, the proposed OHC&RP-SGKD scheme 1 resolves the collusion attack problem.
(2). Reducing the communication redundancy
  Considering that there may have no new joined users in some sessions in real network environments and introducing the unique identity for each session, novel methods are presented to construct the revocation polynomials and the key updating broadcast packet in the proposed OHC&RP-SGKD scheme 1.
  In the proposed OHC&RP-SGKD scheme 1, the revocation polynomials for users joined the group in different sessions are constructed in order that a user can be revoked according to its joining time. And if there are no users joined in session *j′* (*j′* ≤ *j*), $R_{j}^{j^{\prime }}=\emptyset$, $A_{j}^{j^{\prime }}(x)=\emptyset$, and $\Phi_{j}^{j^{\prime }}(x)$ is not included in *B_j_*.
  Suppose that during *j* sessions, the group member addition operation occurs *v* times. The size of the *j*-th key updating broadcast packet, *B_j_*, in the proposed OHC&RP-SGKD scheme 1 and Dutta *et al.*'s scheme is [(*t* + 1)*v* + *j*)]log_2_*q* bits and [(*t* + 1)*j*]log_2_*q* bits, respectively. When *v* < *j*, the size of *B_j_* in the proposed OHC&RP-SGKD scheme 1 is smaller than that of Dutta *et al.*'s scheme.
  Hence, with novel structures of the revocation polynomials and the key updating broadcast packet, the communication redundancy reduces.
(3). Updating of personal secrets partially
  In existing RP-SGKD schemes, once *m* sessions expires or *t* revoked users reaches, these schemes should be reset, and the GM has to update the personal secrets of all legitimate group members because the same personal secret polynomial is shared. In the proposed OHC&RP-SGKD scheme 1, users joined the group in different sessions share different personal secret polynomials, and only the number of revoked users joined the group in the same session reaches *t*, the scheme will be reset. For example, if $|R_{j}^{j'}|=t$ in session *j*, and *j* < *m*, the GM only needs to update the personal secrets of legitimate users in $G_{j}^{j^{\prime }}$.
  Hence, the proposed OHC&RP-SGKD scheme 1 can update the personal secrets partially, which in turn prolongs the lifetime of the scheme.
(4). Eliminating the limitation of *m* < *t* + 1
  In the proposed OHC-RP-SGKD scheme 1, users joined the group in different sessions are treated by binding the joining time with the capability of recovering previous session keys, and they are classified according to the joining time. Users joined the group in different sessions are allocated different shares of personal secret polynomials, which makes users joined the group in different sessions be unable to collude together.

The reset of the SGKD scheme is triggered by two conditions as follows.

CON1: The maximum number of sessions expires although the number of revoked users is less than *t*.

CON2: The number of revoked users reaches *t* although the maximum number of sessions does not expire.

Considering the CON2 that $|{\text{R}}_{j}|=\overset{j}{\underset{j'=1}{\text{∑}}}\left|R_{j}^{j'}\right|\geq t$ in session *j*, *j* < *m* and $|R_{j}^{j'}|<t$. In the proposed OHC&RP-SGKD scheme 1, since users joined the group in different sessions cannot coalesce together, the session key(s) cannot be deduced even if *t* + 1 users joined the group in different sessions are revoked. Hence, the proposed OHC&RP-SGKD scheme 1 does not need to reset.

Hence, the proposed OHC&RP-SGKD scheme 1 can support more sessions under same conditions compared to existing HC-SGKD schemes, and a smaller *t* can be used to prolong the lifetime of the scheme.

#### 3.1.3. Security Analysis

Based on the security model in Section 2, the proposed OHC&RP-SGKD scheme 1 is secure with following theorems and proofs.

##### Theorem 1

*The scheme presented in Section 3.1.1 is a secure, self-healing group session key distribution scheme with mt-revocation capability*.

###### Proof

(a) A legitimate group member *U_i_*, $U_{i}\epsilon G_{j}^{j^{\prime }}$ and *j′* ≤ *j*, can recover *K_j_* as described in Phase 3. Hence, it follows that *H*(*K_j_*|*B_j_*, *S_i_*) = 0.
(b) Since *K_j_* is independent of *S_i_*, using the personal secret only does not give any information about the session keys. On the other hand, since the masking key and the session key are selected randomly, the key updating broadcast packets cannot give any information about the session keys. Therefore, *K_j_* cannot be determined only with *S_j_* or *B_j_*. Hence, it follows that *H*(*K_j_*|*S*_1_, *S*_2_, …, *S_N_*) = *H*(*K_j_*|*B*_1_, *B*_2_, …, *B_m_*) = *H*(*K_j_*).
(c) For $U_{r}\in R_{j}^{j^{\prime }}$, $A_{j}^{j^{\prime }}(r)=0$, which makes $k_{j}^{j'}$ appears randomly to users in $R_{j}^{j'}$. Hence, it is impossible for the coalition of users in **R***_j_* to recover *K_j_* because **R***_j_* has no information about $k_{j}^{j'}$.
  Moreover, since only users joined the group in the same session can coalesce together, the coalition of users joined the group in different sessions cannot get information about å*_j′_*·*s_j_*(*x*). Because $|R_{j}^{j'}|\leq t$, and the required number of users to determine å*_j′_*·*s_j_*(*x*) is at least (*t* + 1), the coalition of users in **R***_j_* cannot recover *å_j′_*·*s_j_*(*x*), which makes *K_j_* appear randomly to all users in **R***_j_*.
  Hence, it follows that *H*(*K_j_|B_j_*, *S_i_*) = 0, *H*(*K_j_*|*B_j_*, {*S_r_*|*U_r_* ∈ **R***_j_*}) = *H*(*K_j_*).
(d) From Phase 3, we observe that the proposed OHC&RP-SGKD scheme 1 makes a user recover lost session keys in previous sessions with current key updating broadcast packet only if the user is not revoked in these sessions.
  Specifically, let *U_i_* who joined the group in session *j*_1_ be a legitimate group member in session *j*_2_, and $U_{i}\in G_{j_{2}}^{j_{1}}$. *U_i_* receives *B_j_*__2__, but not *B_j_*, and *j*_1_ < *j* < *j*_2_. *U_i_* recovers all of the lost session keys as follows.
  (1) In Phase 3, *U_i_*, $U_{i}\epsilon G_{j_{2}}^{j_{1}}$ and *j*_1_ < *j*_2_, recovers $k_{j_{2}}^{j_{1}}$.
  (2) With $k_{j_{2}}^{j_{1}}$, *U_i_* generates all masking keys, $\left\{k_{j_{2}}^{j}|j_{1}<j<j_{2}\right\}$, in the *j*_2_-th one-way hash key chain.
  (3) *U_i_* recovers {*K_j_*| *j*_1_ < *j* < *j*_2_} by decrypting $\left\{E_{k_{j_{2}}^{j}}(K_{j})|j_{1}<j<j_{2}\right\}$ with $\left\{k_{j_{2}}^{j}|j_{1}<j<j_{2}\right\}$.
    Hence, the proposed OHC-RP-SGKD scheme 1 has the property of self-healing. It follows that
    $$H\left(K_{j}|B_{j_{2}},\left\{S_{i}|U_{i}\in G_{j}^{j_{1}}\right\}\right)=0$$

##### Theorem 2

*The scheme presented in Section 3.1.1 achieves mt-wise forward secrecy*.

###### Proof

For $U_{r}\epsilon R_{j}^{j^{\prime }}$, $A_{j+1}^{j^{\prime }}(r)=0$, which means that *U_r_* cannot recover $k_{j+1}^{j^{\prime }}$ unless *U_r_* can guess $k_{j+1}^{j^{\prime }}$ correctly.

Since $\left|R_{j}^{j^{\prime }}\right|\leq t$, å*_j′_*·*_Sj_*_+1_(*x*) cannot be determined by the coalition of users in $R_{j}^{j^{\prime }}$. Moreover, since only users who joined the group in the same session can coalesce together, the coalition of users joined the group in different sessions cannot get information about *å_j′_*·*_Sj_*_+1_(*x*). Hence, although all revoked users in **R***_j_* coalesce together, *å_j′_*·*_Sj_*_+1_(*x*) still cannot be determined, and *K_j_*_+1_ cannot be recovered.

Therefore, the proposed OHC-RP-SGKD scheme 1 is *mt*-wise forward secret. It follows that

$$H\left(K_{j+1}|B_{1},B_{2},\ldots ,B_{m},\left\{S_{r}|U_{r}\epsilon {\text{R}}_{j}\right\},K_{1},K_{2},\ldots ,K_{j}\right)=H\left(K_{j+1}\right)$$

##### Theorem 3

*The scheme presented in Section 3.1.1 achieves any-wise backward secrecy*.

###### Proof

In order to recover *K_j_*, any user *U_i_*, *U_i_* ϵ **D***_j_*, requires the knowledge of at least (*t* + 1) distinct points about å*_j″_*·*_Sj_*(*x*), *j″* ≤ *j*. Suppose that *U_i_* joins the group in session *j′*, the GM gives the personal secret, *S_i_* = {å*_j′_*·*_Sj_*_1_(*i*)| *j* + 1 ≤ *j′* ≤ *j*_1_ ≤ *m*} to *U_i_*. Hence, the coalition of user in **D***_j_* cannot compute å*_j″_*·*_Sj_*(*x*) no matter how many users in **D***_j_*.

Therefore, the proposed OHC-RP-SGKD scheme 1 is *any*-wise backward secret. It follows that

$$H\left(K_{j}|B_{1},B_{2},\ldots ,B_{m},\left\{S_{i}|U_{i}\epsilon {\text{D}}_{j}\right\},K_{j+1},K_{j+2},\ldots ,K_{m}\right)=H\left(K_{j}\right)$$

##### Theorem 4

*The scheme presented in Section 3.1.1 has mt-collusion attack resistance capability*.

###### Proof

Let **R***_j_*_1_ be a set of users be revoked before and in session *j*_1_, **D***_j_*_2_ be the set of users joined the group after session *j*_2_, and *j*_1_ < *j*_2_. We will prove that users in **R***_j_*_1_ colluding with users in **D***_j_*_2_ cannot recover *K_j_* (*j*_1_ < *j* ≤ *j*_2_) with *B_j_*_1_ and *B_j_*_2_.

From Theorem 2, the coalition of users in **R***_j_*_1_ cannot recover *K_j_* for *j* > *j*_1_. Similarly, from Theorem 3, the coalition of users in **D***_j_*_2_ cannot recover *K_j_* for *j* ≤ *j*_2_.

On the other hand, any user *U_r_* in $R_{j_{1}}^{j^{\prime }}$ only knows {*å_j′_*·*_Sj_*(*r*)| *j* ≥ *j′*}, And any user *U_i_* in *D^j″^* only knows {*å_j″_*·*_Sj_*(*i*)| *j* > *j″*}. Since only users joined the group in the same session can coalesce together, users in **R***_j_*_1_ colluding with users in **D***_j_*_2_ obtain no information about *å_j′_*·*_Sj_*(*x*) or *å_j″_*·*_Sj_*(*x*), *j*_1_ < *j* ≤ *j*_2_. Hence, the collusion of users in **R***_j_*_1_ and **D***_j_*_2_ cannot recover *K_j_*, *j*_1_ < *j* ≤ *j*_2_.

Therefore, the proposed OHC-RP-SGKD scheme 1 resists to *mt-wise* collusion attack. It follows that

$$H\left(K_{j}|B_{1},B_{2},\ldots ,B_{m},\left\{S_{i}|U_{i}\in {\text{R}}_{j_{1}}\cup {\text{D}}_{j_{2}}\right\}\right)=H\left(K_{j}\right)$$

### 3.2. The OHC&RP-SGKD Scheme 2

Several parameters have been considered to evaluate the performance of SGKD schemes. With respect to the storage overhead, the proposed OHC-RP-SGKD scheme 1 is not optimal. How to tradeoff among the maximum allowed number of sessions, the maximum allowed number of revoked users, the storage overhead and the communication overhead is still an open issue for the RP-SGKD schemes.

By analyzing the key updating broadcast packet in the proposed OHC-RP-SGKD scheme 1, we observe that each $k_{j}^{j^{\prime }}$ is masked by different masking polynomials, {ε*_j′_*· *s_j_* (*x*) | *j* = *j′*, *j′*+1,…,*m*}. Although using multiple masking polynomials seems to make the attack be more difficult, it does not contribute to the security. Indeed, using one masking polynomial for each $k_{j}^{j^{\prime }}$ is sufficient. Hence, the number of masking polynomials and the personal secret stored by each user reduce.

Based on the above discussion, an OHC&RP-SGKD scheme with a constant storage overhead is proposed, name as the OHC&RP-SGKD scheme 2.

The proposed OHC&RP-SGKD scheme 2, including three phases and two cases, is described as follows.

**Phase 1′: Initialization**

The GM randomly chooses a 2*t*-degree polynomial, *s*_1_(*x*) = *a*_0_ + *a*_1_*x* + … + *a*_2_*_t_x*^2^*^t^*, a *t*-degree polynomial, *s*_2_(*x*) = *b*_0_ + *b*_1_*x* + … + *b_t_x^t^*, from *F_q_*[*x*], and a number, å_1_, from *F_q_*.

Any user *U_i_* in **G_1_** receives the personal secret *S_i_* = {å_1_·*s*_1_(*i*), å_1_·*s*_2_(*i*)} from the GM via a secure communication channel.

**Phase 2′: Broadcast in Session *j* (1 ≤ *j* ≤ *m*)**

The GM randomly chooses a session key *K_j_* and a number $k_{j}^{0}$ from *F_q_*.

The *j*-th key chain, $\left\{k_{j}^{1},k_{j}^{2},\ldots ,k_{j}^{j}\right\}$, is computed with (8). And the GM splits $k_{j}^{j^{\prime }}$ into two *t*-degree polynomials, $U_{j}^{j^{\prime }}(x)$ and $V_{j}^{j^{\prime }}(x)$, in order that

(13) $$k_{j}^{j^{\prime }}=U_{j}^{j^{\prime }}(x)+V_{j}^{j^{\prime }}(x),j^{\prime }=1,2,\ldots ,j$$

The GM constructs and broadcasts the message

(14) $${Β}_{j}={\text{R}}_{j}\cup {{\text{R}}^{\prime }}_{j}\cup \left\{M_{j}^{j^{\prime }}(x)|j^{\prime }=1,2,\ldots ,j\right\}\cup \left\{N_{j}^{j^{\prime }}(x)|j^{\prime }=1,2,\ldots ,j\right\}\cup \left\{E_{k_{j}^{j^{\prime }}}\left(K_{j^{\prime }}\right)|j^{\prime }=1,2,\ldots ,j\right\}$$

where

(15) $$M_{j}^{j^{\prime }}(x)=A_{j}^{j^{\prime }}(x)\cdot U_{j}^{j^{\prime }}(x)+\varepsilon_{j^{\prime }}\cdot s_{1}(x)$$

(16) $$N_{j}^{j^{\prime }}(x)=V_{j}^{j^{\prime }}(x)+\varepsilon_{j^{\prime }}\cdot s_{2}(x)$$

The definitions of **R***_j_*, **R′***_j_* and the structure of revoked polynomials, $\left\{A_{j}^{j^{\prime }}(x)|j^{\prime }=1,2,\ldots ,j\right\}$, are the same as those in Phase 2 of the proposed OHC&RP-SGKD scheme 1.

**Phase 3′: Group Session Key Recovery in Session *j* (1 ≤ *j* ≤ *m*)**

Any legitimate group member *U_i_* in $G_{j}^{j^{\prime }}\left(j^{\prime }\leq j\right)$ can recover the group session key from *B_j_* through following steps.

(1) *U_i_* computes $U_{j}^{j^{\prime }}(i)=\left[M_{j}^{j^{\prime }}(i)-\varepsilon_{j^{\prime }}\cdot s_{1}(i)\right]/A_{j}^{j^{\prime }}(i)$ and $V_{j}^{j^{\prime }}(i)=N_{j}^{j^{\prime }}(i)-\varepsilon_{j^{\prime }}\cdot s_{2}(i)$ with (15) and (16), respectively. Thus, $k_{j}^{j^{\prime }}=U_{j}^{j^{\prime }}(i)+V_{j}^{j^{\prime }}(i)$.
(2) *U_i_* computes all of the remaining keys in the *j*-th key chain, $\left\{k_{j}^{j^{\prime \prime }}|j^{\prime }<j^{\prime \prime }\leq j\right\}$.
(3) By decrypting $\left\{E_{k_{j}^{j^{\prime \prime }}}\left(K_{j^{\prime \prime }}\right)|j^{\prime }<j^{\prime \prime }\leq j\right\}$ with $\left\{k_{j}^{j^{\prime \prime }}|j^{\prime }<j^{\prime \prime }\leq j\right\}$, *U_i_* recovers {*k_j″_*| *j′* < *j″* ≤ *j*}.

**Case 1′: Group Member Addition**

When a new user, *U_v_*, joins the group in session *j*, the GM allocates *S_v_* = {å*_j_*·*s*_1_(*v*), å*_j_*·*s*_2_(*v*)} to it via the secure communication channel. Receiving the personal secret, *U_v_* joins **G***_j_*.

The GM and users in **G***_j_* launch a key updating process, including Phase 2′ and Phase 3′, to include *U_v_*.

**Case 2′: Group Member Revocation**

The operation of group member revocation is the same as that described in the Case 2 of the proposed OHC&RP-SGKD scheme 1.

The proposed OHC&RP-SGKD scheme 2 holds all of the advantages described in Section 3.1.2, and also has constant storage overhead for the personal secret of each user.

Along the same lines of the proof of Theorems 1–4, we have the Theorem 5 as follows.

#### Theorem 5

*The scheme presented in Section 3.2.1 is a secure, self-healing key distribution scheme with mt-revocation capability, and achieves mt-wise forward secrecy, any-wise backward secrecy, and mt-wise collusion attack resistance capability*.

## 4. Performance Analysis and Comparisons

The performance comparison, in terms of the storage overhead, the communication overhead, the computation overhead, the forward secrecy, the backward secrecy and the collusion attack resistance capability, is listed in Table 1.

### 4.1. The Storage Overhead for the Personal Secret

The storage overhead for the personal secret of each user comes from the initialization phase. In the proposed OHC&RP-SGKD scheme 1, the storage overhead for the personal secret of each user is (*m* − *j* + 1)log_2_*q* bits, which is as same as that of schemes in [5,7,8].

In the proposed OHC&RP-SGKD scheme 2, the storage overhead for the personal secret of each user is 2log_2_*q* bits, which is independent of *m* and *t*, and much less than that of the proposed OHC&RP-SGKD scheme 1 and other existing schemes in [4–8,10,11,20].

### 4.2. The Communication Overhead for Updating Session Keys

The communication overhead for updating session keys comes from *B_j_*. In the proposed OHC&RP-SGKD scheme 1, if there are no users joined in session *j′*, $\Phi_{j}^{j'}\left(x\right)$ is not included in *B_j_*. Suppose that the joining operation occurs *v* times during *j* sessions, *B_j_* consists of a set of revoked users **R***_j_*, **R**′*_j_*, *v t*-degree polynomials, $\left\{\Phi_{j}^{j'}(x)\right\}$, and the sequence, $\left\{E_{k_{j}^{j'}}\left(K_{j'}\right)|j'=1,2,\ldots ,j\right\}$. The communication overhead for broadcasting **R***_j_* and **R**′*_j_* can be ignored because the IDs can be selected from a small finite field [7]. Hence, the size of *B_j_* is about [(*t* + 1)*v* + *j*]log_2_*q* bits, which is the same as that of the RP-SGKD scheme in [20], and less than that of existing schemes in [4–8,11],where *v* < *j* ≤ *m*.

In the proposed OHC&RP-SGKD scheme 2, the size of *B_j_* is [(3*t* + 2)*v* + *j*]log_2_*q* bits, which is larger than that of the proposed OHC&RP-SGKD scheme 1.

**Figure 1.**
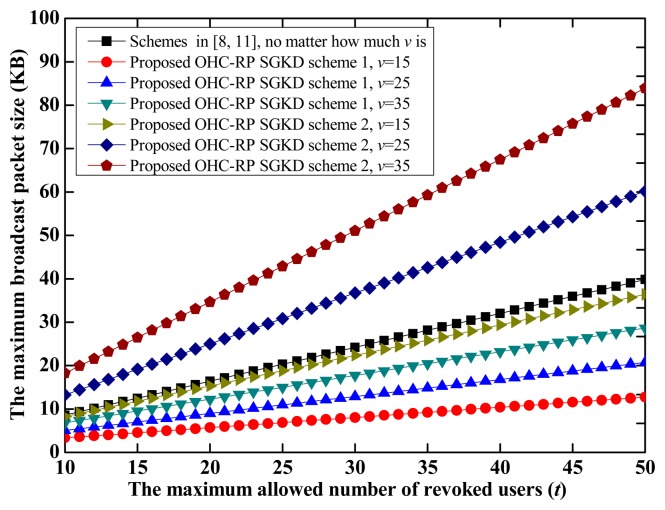
The comparison of the maximum broadcast packet size.

As the assumption in [13], the maximum number of sessions is set to be *m* = 50. Figure 1 shows the comparison of the maximum broadcast packet size when *t* varies from 10 to 50. Without loss of generality, *q* is set to be a 128-bit integer.

From Figure 1, we observe that, when *v* < *m*, the size of *B_j_* in the proposed OHC&RP-SGKD scheme 1 is smaller than that of schemes in [8,11] and with the same *m* and *t*. For example, when *m* = 50 and *t* = 50, the broadcast packet sizes of the proposed OHC&RP-SGKD scheme 1 are about 12.734 KB, 20.703 KB, and 28.671 KB for *v* = 15, 25 and 35, respectively, while the broadcast packet size of schemes in [8,11] is about 39.844 KB. Moreover, the maximum broadcast packet size in the proposed OHC&RP-SGKD scheme 2 is obviously larger than that of the proposed OHC&RP-SGKD scheme 1, especially is larger than that of schemes in [8,11].

***Remark:*** It is necessary to reduce the communication redundancy as possible. Although the communication overhead in the proposed OHC&RP-SGKD scheme 1 increases with the number of sessions, it grows more slowly than that of schemes in [8,11] under same conditions.

On the other hand, although the broadcast packet size of the proposed OHC&RP-SGKD scheme 2 is larger than that of the proposed OHC&RP-SGKD scheme 1, we will prove later that the total communication overhead for updating group session keys and the personal secrets in the proposed OHC&RP-SGKD scheme 2 is smaller.

### 4.3. Practicality

Many practical issues should be addressed when an SGKD scheme is implemented in a real-world application.

As we know, ZigBee, a protocol designed for low data rate wireless networks, uses the IEEE 802.15.4 physical and MAC layers to provide data transfer. According to the IEEE 802.15.4 protocol [31], the maximum size of MAC layer payload is from 89 to 119 bytes. When the maximum size of MAC layer payload is 89 bytes, the application layer data larger than 89 bytes will be partitioned into blocks.

Due to the unreliable wireless transmission, the maximum broadcast packet size in the SGKD scheme is also limited. Let the maximum broadcast packet size be 4096 bytes (4 KB), which will be partitioned into 46 packets with 89 bytes/packet. If packets are lost independently and randomly at a rate of 1%, the probability that a 4 KB broadcast packet will not reach the destination is 37.01%. If the packet loss rate is 5% (a fairly high), the probability that a 4 KB broadcast packet reaches the destination is only 9.45%. Hence, *m* should be larger than 10. However, the maximum broadcast packet size is assumed to be 64 KB in most existing SGKD schemes [4–7], which is not applicable in ZigBee-based wireless networks.

**Figure 2.**
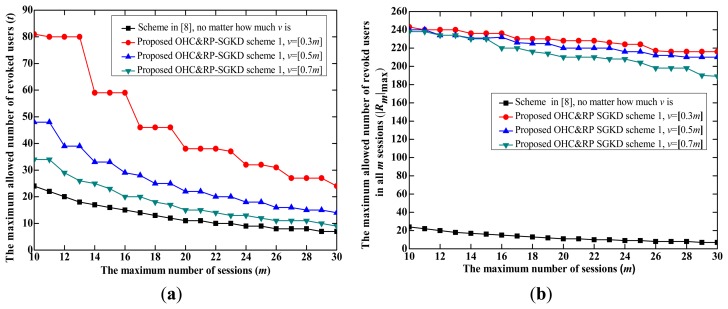
The performance comparison between the proposed one-way hash chain and revocation polynomial-based self-healing group key distribution (OHC&RP-SGKD) scheme 1 and the scheme in [8]. (**a**) The tradeoff between *m* and *t*; (**b**) The tradeoff between *m* and |R*_m_*|_max_.

**Figure 3.**
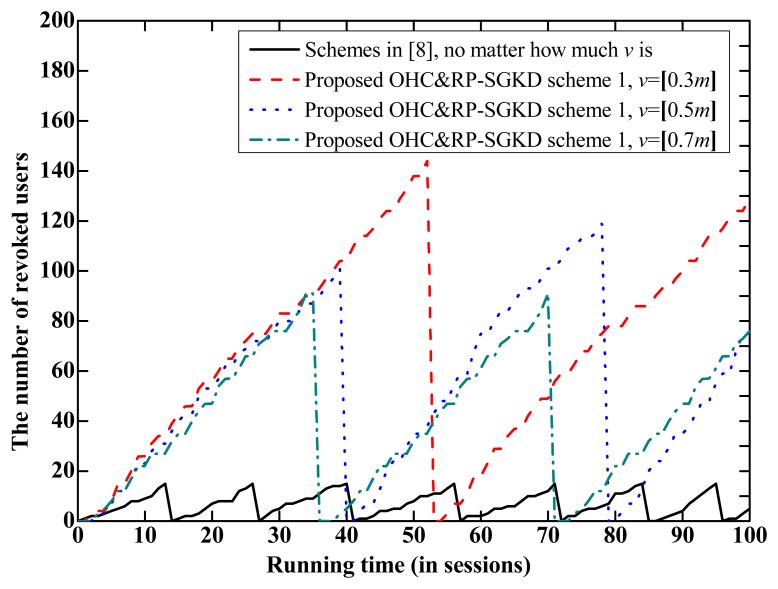
The possible lifetime in 100 sessions.

**Figure 4.**
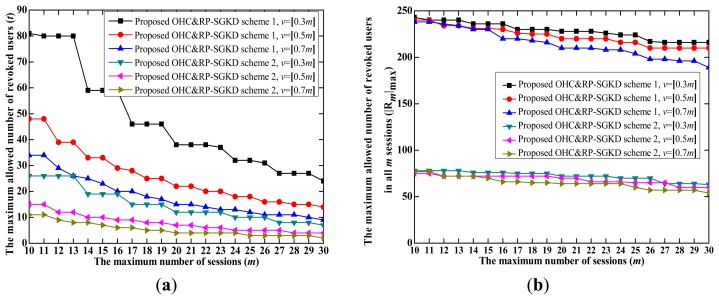
The performance comparison of the proposed one-way hash chain and revocation polynomial-based self-healing group key distribution (OHC&RP-SGKD) schemes 1 and 2. (**a**) The tradeoff between *m* and *t*; (**b**) The tradeoff between *m* and |R*_m_*|_max_.

With the limitation of the maximum broadcast packet size, the value of other parameters should be appropriately set for the intended application and compatible with existing network protocols. In SGKD schemes, system parameters affecting the broadcast packet size are the number of sessions (*m*), the size of the session key (log_2_*q*), and the degree of the personal polynomial (*t*). Without loss of generality, it is assumed that *q* is a 128-bit integer, and session keys are also 128 bits, which are used in a symmetric cipher, such as AES. The maximum broadcast packet size is set to be 4KB. Symbol [*x*] represents the operation to round *x* to the integer downward.

(1). The proposed OHC&RP-SGKD scheme 1 *vs.* the scheme in [8]
  The performance of the proposed OHC&RP-SGKD scheme 1 is compared to that of the scheme in [5] because the storage overhead of each user in these two schemes is same, both of them are the RP-SGKD schemes, and the scheme in [8] is the best one among existing collusion-attack-resistance schemes in [4–8]. Let |**R***_m_*|_max_ be the maximum allowed number of revoked users in *m* sessions.
  Figure 2 shows performance comparison between the proposed OHC&RP-SGKD scheme 1 and the scheme in [8], where Figure 2a is the tradeoff between *m* and *t*, and Figure 2b is the tradeoff between *m* and |**R***_m_*|_max_.
  From Figure 2a, we observe that the proposed OHC&RP-SGKD scheme 1 can support more sessions than the scheme in [8]. In the proposed OHC&RP-SGKD scheme 1, a smaller *t* can be used to prolong the lifetime of the scheme because users joined the group in different sessions cannot coalesce together. For example, when *t* = 15 and *m* = 16, |**R***_m_*|_max_ = 15 for the scheme in [8], whereas for the proposed OHC&RP-SGKD scheme 1, when *t* = 15, *m* = 44, 28 and 20, |**R***_m_*|_max_ = 195, 210 and 210 for *v* = 0.3 *m*, 0.5 *m* and 0.7 *m*, respectively. And when *t* = 10, *m* = 59, 39 and 29, |**R***_m_*|_max_=170, 190 and 200 for *v* = 0.3 *m*, 0.5 *m* and 0.7 *m*, respectively.
  Moreover, the proposed OHC&RP-SGKD scheme 1 can revoke much more users than that of the scheme in [8]. For example, from Figure 2b, when *m* = 20, |**R***_m_*|_max_ = 11 for the scheme in [8], whereas |**R***_m_*|_max_ = 210, 220 and 232 for *v* = 0.7 *m*, 0.5 *m* and 0.3 *m*, respectively, in the proposed OHC&RP-SGKD scheme 1. Obviously, the proposed OHC&RP-SGKD scheme 1 allows much more revoked users and withstands much more colluding users compared to the scheme in [8].
  In a real-world application, the longer the scheme runs, the more users are revoked. Figure 3 shows the possible lifetime of the proposed OHC&RP-SGKD scheme 1 and the scheme in [8] when two schemes are simulated during 100 sessions.
  From Figure 3, we observe that with small values of *m* and *t*, the scheme in [8] will be reset frequently, which leads to the energy and bandwidth consumption. However, in the proposed OHC&RP-SGKD scheme 1, more revoked users and more sessions are allowed, and less resetting of the proposed OHC&RP-SGKD scheme 1 contributes to saving the network energy.
  Therefore, the advantage of the proposed OHC&RP-SGKD scheme 1 is obvious for ZigBee-based wireless networks in bad environment where a strong collusion attack resistance is required and many users need to be revoked.
(2). The proposed OHC&RP-SGKD scheme 2 *vs.* the proposed OHC&RP-SGKD scheme 1
  In the proposed OHC&RP-SGKD scheme 1 and other existing RP-SGKD schemes, since the storage overhead at each user increases along with the increase of *m* or *t*, the power and bandwidth consumption for re-keying personal secrets will be much large. However, the proposed OHC&RP-SGKD scheme 2 has constant storage overhead of 2log_2_*q* bits.
  Figure 4 show the performance comparison of the proposed OHC&RP-SGKD schemes 1 and 2, where Figure 4a is the tradeoff between *m* and *t*, and Figure 4b is the tradeoff between *m* and |**R***_m_*|_max_.
  From Figure 4a,b, we observe that the values of *t* and *m* in the proposed OHC&RP-SGKD scheme 2 are smaller than those of the proposed OHC&RP-SGKD scheme 1 under same conditions. However, since the storage overhead for each user in the proposed OHC&RP-SGKD scheme 2 is much less than that of the proposed OHC&RP-SGKD scheme 1, the communication overhead for rekeying the personal secrets in the proposed OHC&RP-SGKD scheme 2 is much less than that in the proposed OHC&RP-SGKD scheme 1.
  Wireless devices are usually powered by battery, and most energy is consumed by the communication module. The main concern of the proposed OHC&RP-SGKD scheme 2 is to reduce the total communication overhead for updating the personal secrets and session keys.
  Suppose that *n* users maintain membership during *m* sessions. For the proposed OHC&RP-SGKD scheme 1, the communication overhead for distributing the personal secrets to *n* users is *nm*log_2_*q* bits in the initialization phase, and the communication overhead for updating session keys is [(*t* + 1)*v* + *j*]log_2_*q* bits in the broadcast phase. After running *m* sessions, the scheme will be reset and new personal secrets should be re-allocated to each group member. Hence, the total communication overhead for updating session keys and the personal secrets of *n* users in the proposed OHC&RP-SGKD scheme 1 is
  (17) $$E^{\left(1\right)}=\left\{nm^{\left(1\right)}+\sum_{j=1}^{m^{\left(1\right)}}\left[\left(t^{\left(1\right)}+1\right)v+j\right]\right\}\log_{2}q(\text{bits})$$
  where, *m*^(1)^ and *t*^(1)^ denote the session number and the number of revoked users when the proposed OHC&RP-SGKD scheme 1 is reset, respectively.
  In the proposed OHC&RP-SGKD scheme 2, the communication overhead for distributing the personal secrets to *n* users is 2*n*log_2_*q* bits, and the communication overhead for updating session keys is [(3*t* + 2)*v* + *j*]log_2_*q* bits. Thus, the total communication overhead is
  (18) $$E^{\left(2\right)}=\left\{2n+\sum_{j=1}^{m^{\left(2\right)}}\left[(3t^{\left(2\right)}+2)v+j\right]\right\}\log_{2}q(\text{bits})$$
  where, *m*^(2)^ and *t*^(2)^ denote the session number and the number of revoked users when the proposed OHC&RP-SGKD scheme 2 is reset, respectively.
  According to the results of Figure 4, when *v* = 0.5 *m*, *m*^(1)^ = 22, *t*^(1)^ = 20, *m*^(2)^ = 14, *t*^(2)^ = 10. Hence, after running 154 sessions, the proposed OHC&RP-SGKD scheme 1 is reset seven times and the proposed OHC&RP-SGKD scheme 2 is reset 11 times. Hence, during the 154 sessions, the decrement of the total communication overhead for updating session keys and the personal secrets in the proposed OHC&RP-SGKD schemes 1 and 2 is *ΔE* = *E*^(1)^ − *E*^(2)^ = 232.72 KB when *n* = 100.
  Hence, the proposed OHC&RP-SGKD scheme 2 has less storage and total communication overheads, and is therefore quite suitable for resource-constrained wireless networks.

## 5. Conclusions

To solve the collusion attack problem in existing HC-SGKD schemes, eliminate the limitation of the maximum allowed number of revoked users on the maximum allowed number of sessions, and improve the security and efficiency of existing RP-SGKD schemes, we proposed two improved SGKD schemes using the one-way hash chain and the revocation polynomial for resource-constrained wireless networks in this paper. In the proposed OHC&RP-SGKD schemes, by introducing the unique session identifier and binding the joining time with the capability for recovering previous session keys, the problem of the collusion attack between revoked and new joined users in existing HC-SGKD schemes is resolved. And novel methods for utilizing the one-way hash chain and constructing the personal secret, the revocation polynomial and the key updating broadcast packet are presented to eliminate of the limitation of the maximum allowed number of revoked users on the maximum allowed number of sessions, increase the maximum allowed number of revoked users, and reduce the redundancy in the key updating broadcast packet.

With the security and performance analysis, we concluded the proposed improved OHC&RP-SGKD schemes as follows.

(1) In the proposed OHC&RP-SGKD scheme 1, the impact of *t* on *m* is eliminated and the maximum allowed number of sessions is enlarged. In the proposed OHC&RP-SGKD scheme 2, the storage overhead for the personal secret in each user is constant, 2log_2_*q* bits, and a better tradeoff between the storage overhead and the total communication overhead is also achieved.
(2) Two proposed improved OHC&RP-SGKD schemes are secure, achieve *mt*-revocation capability, *mt*-wise forward secrecy, *any*-wise backward secrecy, and *mt*-wise collusion attack resistance capability.
(3) The communication overhead of the proposed OHC&RP-SGKD schemes is lower compared to existing RP-SGKD schemes.
(4) Simulation results show that the proposed OHC&RP-SGKD schemes are practical for resource-constrained wireless networks in bad environments where a strong collusion attack resistance is required and many users should be revoked.

For an SGKD scheme, a challenging problem is how to achieve a better tradeoff between the storage overhead and the communication overhead. Since the key updating broadcast packet in the proposed OHC&RP-SGKD scheme 2 is still large, we will focus on reducing the communication overhead in the future work.

## Figures and Tables

**Table 1.** Performance comparison results.

**Schemes**	**Storage Overhead for Personal Secret (Bits)**	**Communication Overhead for Updating Session Keys (Bits)**	**Computation Overhead (the Number of Multiplication Operations)**	**Forward Secrecy**	**Backward Secrecy**	**Collusion Attack Resistance**
Scheme 3 in [4]	(*m* − *j* + 1)^2^log_2_*q*	(*mt*^2^ + 2*mt* + *m* + *t*)log_2_*q*	2*mt*^2^ + 3*mt* − *t*	Yes/*t*	Yes/*t*	Yes/*t*
Scheme 2 in [5]	(*m* − *j* + 1)log_2_*q*	(*jt*^2^+ *jt*)log_2_*q*	(2*t* + 1)(*m* + 1)	Yes/*t*	Yes/*t*	Yes/*t*
Scheme 3 in [6]	2(*m* − *j* + 1)log_2_*q*	[(*m* + *j* + 1)*t* + (*m* + 1)]log_2_*q*	*mt* + *t* + 2*tj* + *j*	Yes/*t*	Yes/*t*	Yes/*t*
Scheme 3 in [7]	(*m* − *j* + 1)log_2_*q*	(2*t* + 1)*j*log_2_*q*	2*j*(*t*^2^ + *t*)	Yes/*t*	Yes/*t*	Yes/*t*
Scheme 2 in [8]	(*m* − *j* + 1)log_2_*q*	(*t* + 1)*j*log_2_*q*	(3*t* + 1)*j*	Yes/*t*	Yes/*t*	Yes/*t*
Scheme in [9]	2log_2_*q*	(*t* + *j* + 1)log_2_*q*	2*t* + 1	No	No	No
Scheme in [10]	(*t* + 2)log_2_*q*	(*t* + 1 + *j*)log_2_*q*	3*t* + 1	Yes/*t*	No	No
Scheme 2 in [11]	(*t* + 2)log_2_*q*	(*t* + 1)*j*log_2_*q*	(3*t* + 1)*j*	Yes/*t*	Yes/*t*	No
Scheme in [20]	(*t* + 2)log_2_*q*	[(*t* + 1)*v* + *j*]log_2_*q*	3*t* + 1	Yes/*mt*	Yes/any	Yes/*mt*
Proposed OHC&RP-SGKD scheme 1	(*m* − *j* + 1)log_2_*q*	[(*t* + 1)*v* + *j*]log_2_*q*	2*t* + 1	Yes/*mt*	Yes/any	Yes/*mt*
Proposed OHC&RP-SGKD scheme 2	2log_2_*q*	[(3*t* + 2)*v* + *j*]log_2_*q*	3*t* + 1	Yes/*mt*	Yes/any	Yes/*mt*

## Appendix

**Table A1.** Notations.

**Notations**	**Denotations**
*U_i_*	the *i*-th user
*N*	the total number of users in a communication group
*m*	the maximum allowed number of sessions
*t*	the maximum allowed number of revoked users
*j*, *j′*, *j″*	the order of a session
*v*	the number of sessions with new joined user(s) during *m* sessions, *v* < *m*
*F_q_*	a finite field of order *q*, and *q* is a prime larger than *N*
*S_i_*	the personal secret of *U_i_*
*B_j_*	the key updating broadcast packet in session *j*
*K_j_*	the session key generated by the GM for session *j*
*H*(*X*)	the entropy of the random variable *X*
*H*(*X|Y*)	the entropy of *X* conditioned on *Y*
*h*(·)	the random one-way function used to compute the one-way key chain
*h^i^*(·)	applying hash operation *i* times
*E_k_*(.)/*D_k_*(.)	a symmetric encryption/decryption function
*å_j_*	the unique session identifier, a random number selected by the GM for users joined the group in session *j*, *å_j_* ∈ *F_q_* and ε*_j_*_1_ ≠ ε*_j_*_2_ for *j*_1_ ≠ *j*_2_
$k_{j}^{0}$	the seed of the *j*-th key chain randomly selected by the GM for session *j*, $k_{j}^{0}\epsilon F_{q}$, and $k_{j_{1}}^{0}\neq k_{j_{2}}^{0}$ for *j*_1_ ≠ *j*_2_
$k_{j}^{j'}$	the *j′*-th key in the *j*-th key chain
$A_{j}^{j'}(x)$	the revoked polynomial constructed by the GM with the IDs of users joined the group in session *j′* and be revoked before or in session *j*, and *j′* ⩽ *j*
$R_{j}^{j'}$	the set of users joined the group in session *j′* and be revoked before or in session *j*, and *j′* ⩽ *j*
$\left|R_{j}^{j'}\right|$	the number of users in $R_{j}^{j'}$
**R***_j_*	the set of users be revoked before and in session *j*, and ${\text{R}}_{j}=\left\{R_{j}^{1},R_{j}^{2},\ldots ,R_{j}^{j}\right\}$
|**R***_j_*|	the number of users in **R***_j_*
*D^j^*	the set of users joined the group in session *j*
**D***_j_*	the set of users joined the group after session *j*, and **D***_j_* = {*D^j^*^+1^, *D^j^*^+2^, …, *D^m^*}
$G_{j}^{j'}$	the set of group members who join the group in session *j′* and are still legitimate in session *j*, and *j′* ⩽ *j*
**G***_j_*	the set of all legitimate group members in session *j*, and ${\text{G}}_{j}=\left\{G_{j}^{1},G_{j}^{2},\ldots ,G_{j}^{j}\right\}$

## Author Conrtibutions

Huifang Chen and Lei Xie proposed two improved OHC&RP-SGKD schemes, analyzed and compared the performance; Lei Xie contributed the simulation results and figures; Huifang Chen wrote the manuscript.
